# Tracking Clinical Staff Behaviors in an Operating Room

**DOI:** 10.3390/s19102287

**Published:** 2019-05-17

**Authors:** Christine Azevedo-Coste, Roger Pissard-Gibollet, Gaelle Toupet, Éric Fleury, Jean-Christophe Lucet, Gabriel Birgand

**Affiliations:** 1 Institut National de Recherche en Informatique et en Automatique (INRIA), Université de Montpellier, 34095 Montpellier, France; 2 Institut National de Recherche en Informatique et en Automatique (INRIA), Grenoble Rhône-Alpes, 38330 Montbonnot, France; roger.pissard@inria.fr (R.P.-G.); eric.fleury@inria.fr (É.F.); 3 APHP Bichat University Hospital, Infection Control Unit, 75018 Paris, France; gaelle.toupet@gmail.com (G.T.); jean-christophe.lucet@bch.aphp.fr (J.-C.L.); gbirgand@gmail.com (G.B.); 4 Institute for Medical Research (INSERM), Infection Antimicrobials Modelling Evolution laboratory (IAME UMR 1137), 75018 Paris, France; 5 Universities Paris Diderot, Infection Antimicrobials Modelling Evolution laboratory (IAME UMR 1137), Sorbonne Paris Cité, 75018 Paris, France

**Keywords:** human behavior analysis, operating room displacements, motion capture, sensor network

## Abstract

Inadequate staff behaviors in an operating room (OR) may lead to environmental contamination and increase the risk of surgical site infection. In order to assess this statement objectively, we have developed an approach to analyze OR staff behaviors using a motion tracking system. The present article introduces a solution for the assessment of individual displacements in the OR by: (1) detecting human presence and quantifying movements using a motion capture (MOCAP) system and (2) observing doors’ movements by means of a wireless network of inertial sensors fixed on the doors and synchronized with the MOCAP system. The system was used in eight health care facilities sites during 30 cardiac and orthopedic surgery interventions. A total of 119 h of data were recorded and analyzed. Three hundred thirty four individual displacements were reconstructed. On average, only 10.6% individual positions could not be reconstructed and were considered undetermined, i.e., the presence in the room of the corresponding staff member could not be determined. The article presents the hardware and software developed together with the obtained reconstruction performances.

## 1. Introduction

Surgical site infection is a major public health problem. Most surgical and infection control societies advise to minimize the traffic in the operating room (OR) in order to decrease air and wound contamination. This includes limiting door opening, movements, and the number of persons inside the room during procedures. To date, these recommendations do not rely on solid scientific proof. Even though, some authors have demonstrated a correlation between airborne contamination and wound contamination [1,2]. The impact of staff behavior on surgical site infection risks was correlated to the number of persons in the OR and the frequency of door opening [3,4,5]. Existing studies were based on audits and human observations. No study has described global staff behaviors during surgical intervention. We have designed a protocol to assess quantitatively and objectively OR staff behaviors and correlate them with the risk of infection during surgical procedures [6]. Indoor localization is still an open problem [7], and the challenge was to develop a least intrusive and mobile indoor localization sensing system to be used in different operating rooms. OR are typically cumbersome environments with the frequent number of ten persons inside the room [8]. The device had to fit with the room configuration without modifying the environment (moving clinical equipment, drilling holes, screwing on the wall or doors, etc.) as is classically done in health smart home studies. The presence of much equipment inside the OR room is also a strong limitation as it may disturb wave propagation and therefore distance estimation using radio frequency-based solutions. The clinical centers participating in the study also requested to not record video due to privacy concerns. All these constraints rule out certain technological choices used in indoor location experiments such as sensing tiles [9], radio-based indoor localization (RFID [10], UWS, ZigBee, Bluetooth [7], and video cameras [11]. In the literature, motion capture (MOCAP) technology was mainly been used to analyze body kinematics, movements, and gestures from a biomechanical perspective [12,13,14]. It was also used for surgical gesture observation and analysis [15], augmented reality [16], and surgeon posture assessment [17,18], usually during simulated surgical procedures. To our knowledge, no previous article has presented the use of an MOCAP system in an operating room for people displacement tracking.

We have conducted an observational multi-center study to assess: door openings, intra-operative staff movements, microbiological air counts, longitudinal particle counts, and bacteriological samples of the wound before skin closure [8]. In the present article, we describe the solution developed to track the door openings and the movements of the staff in the operating room. We present detailed analysis results from data of 30 of the interventions with complete MOCAP and door opening recordings. The innovation is based on the original use and integration of an existing technology.

## 2. Description of the Behavior Assessment System

We have designed a complete setup and methodology to assess continuously the number and category of persons present inside the operating room (OR), their displacements inside the room, and the door opening/closing events. The developed solution integrates an optical MOCAP and wireless inertial sensors. All the equipment required for the study was disinfected daily with a detergent/disinfectant solution before installation, including cameras, cables, markers, and the processing unit. This has been performed in accordance with the World Health Organization guidelines on the prevention of surgical site infection (https://apps.who.int/iris/bitstream/handle/10665/277399/9789241550475-eng.pdf?ua=1, Global Guidelines for the Prevention of Surgical Site Infection, World Health Organisation. 2018).

### 2.1. Motion Capture System

The MOCAP system allows recording the movement of objects or people. An eight-optical camera VICON Bonita^®^ (https://www.vicon.com/products/archived-products/bonita) motion capture system was used together with Tracker^®^ software. These optical systems utilize data captured from image sensors to triangulate the 3D position of a subject between cameras calibrated to provide overlapping projections. Data acquisition was implemented using passive markers, spheres with retro-reflective material, attached to actors or objects.

**Figure 1:**
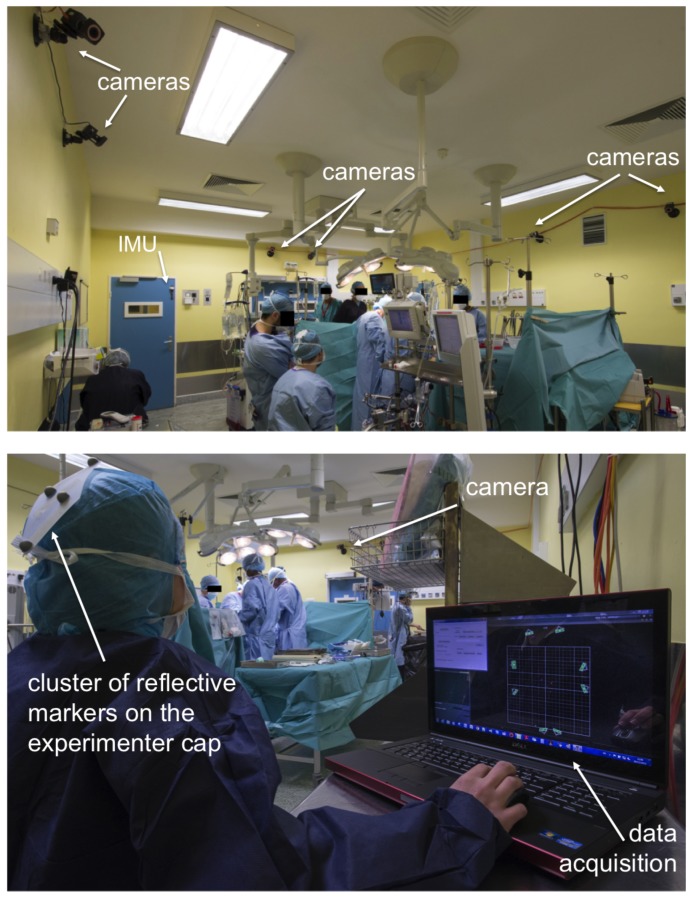
Overview of one session of operating room activity monitoring.

Tracker^®^ allows multiple object tracking and real-time streaming of data (Figure 1). An object was defined by a rigid cluster of reflective markers. We have developed a C++ software in order to manage and acquire data available from the streaming pipeline using the available software development kit (SDK) from VICON. We have recorded the 3D position of the barycenter of the cluster. A user interface allowed the operator to record particular events (inputs/outputs, operations phase, technical incidents, etc.). No video was recorded.

**Figure 2:**
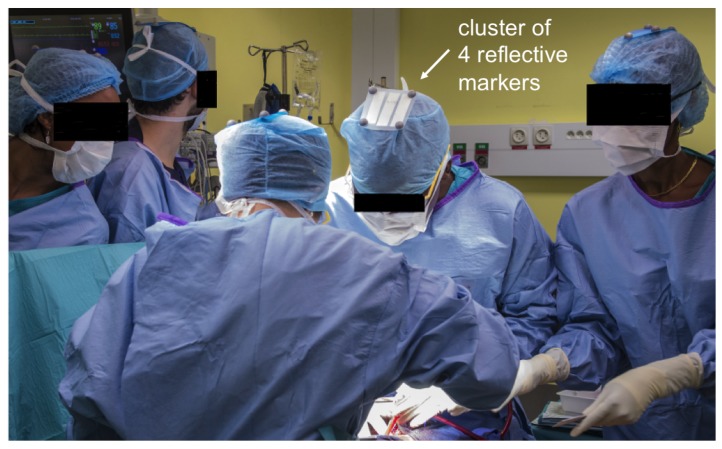
Surgical caps were equipped with clusters of four reflective markers. One arrangement of markers corresponds to one object, the position of which is tracked continuously.

Surgical caps were prepared in advance with light and rigid clusters of four reflective markers (Figure 2). Markers were fixed to the head cover with scotch tape supporting 500 kg and could not be detached without tearing the cover. Each cluster had a specific geometric arrangement of markers and constituted a unique target. A target was associated by one Tracker^®^ object corresponding with one type of staff category: surgeon, anesthetist, nurse, other. Each individual entering the OR was instructed by the experimenter to wear one of the head covers corresponding to his/her category.

Cameras were installed in order to optimize the acquisition volume and avoid occlusion from OR equipment. After proper calibration of the system, the Tracker software was used to detect marker-based models entering the acquisition volume and to track the corresponding objects’ positions in the room.

The environment of an operating room is not ideal for MOCAP acquisition: cameras’ positioning and fastening possibilities are limited; obstacles are numerous and generate reflections; a large number of people can enter and leave the room.

Therefore, in this context, two main issues had to be considered: the existence of ghost markers that should not be confused with true reflective markers; and the occlusion of markers that should not be misinterpreted as a lack of target in the OR. Some ambiguities on the interpretation of the absence of position for an object or the occurrence of outlier positions were resolved by crossing the door opening/closing information with MOCAP object displacements.

### 2.2. Inertial Measurement Units Wireless Network

The technical challenge was to develop a system adaptable to the different operating room configurations without modifying the environment (drill holes, screw sensors on the doors). In order to collect the status of the doors (open/closed) of the OR, we used a data logger system constituted of FOX^®^ sensor nodes (https://www.hikob.com/wp-content/uploads/2015/06/HIKOB_FOX_ProductSheet_EN.pdf). Each node was an inertial measurement unit: compact, low power, and wireless. It included a 3D accelerometer, a 3D gyroscope, and a 3D magnetometer. Although a little oversized to measure door openings, we already had these IMUs and had much experience in their use.

The network of sensor nodes was time synchronized by a radio protocol using the 2.4-GHz radio transceiver, IEEE 802.15.4e standardized. The synchronizer was a dedicated FOX node, which synchronized the other FOX sensors’ clocks. By redundancy, at the beginning of the experiment, before their installation on doors, a “manual” synchronization of the sensors was carried out, by physically grouping them and giving them a shock. This shock recorded by the sensor accelerators at the same time gave a starting reference in case of wireless communication failure. The data storage of sensors nodes was done on an embedded SD card for off-line analysis.

**Figure 3:**
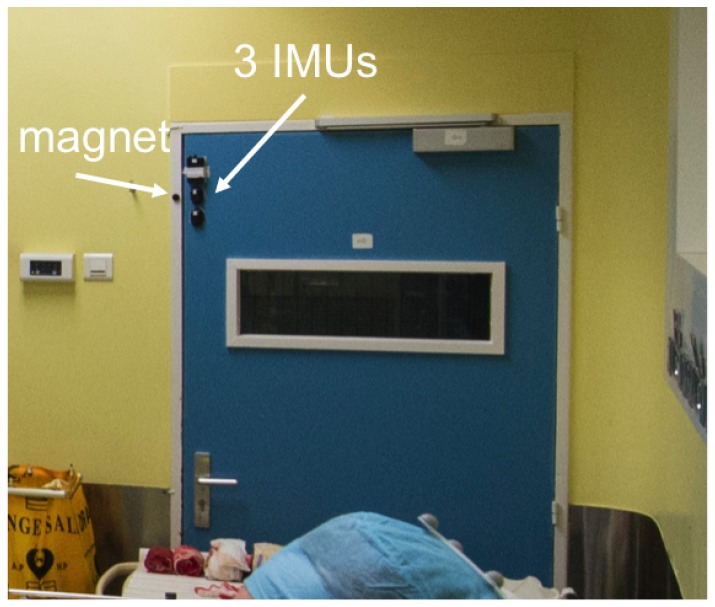
Three inertial measurement units positioned on the left top corner of a door in the operating room.

We placed two sensor nodes on each door (see Figure 3). The aim was to obtain a redundant recording in order to be robust to failures and avoid data loss.

We also attached a magnet on the door-frame nearby the sensor nodes when the door was closed. This provided a magnetometer signal of exaggerated amplitude between a closed door and an open door.

At the beginning of data collection, the software running on a PC master was sending a start clock signal, allowing us to synchronize the multi-point door status acquisition system and the MOCAP system.

Depending on the operating rooms, up to four doors could be equipped, including pivoting and translating opening door types.

The purpose of the deployed system was to quantify, at each time instant, the activity in the operating room through three main pieces of information:

- number of persons in the room,
- 2D position of the persons present in the room,
- door status (open/closed)

### 2.3. Data Acquisition

The two systems, MOCAP and IMU, were synchronized, and data were collected every 200 ms. The calibration of the MOCAP system was done before the arrival of professionals into the operating room and at least one hour before patient entry to reduce the risks of air contamination during calibration. Indeed, the air ventilation system performed a minimum of 25 air renewals of the OR volume per hour.

**Figure 4:**
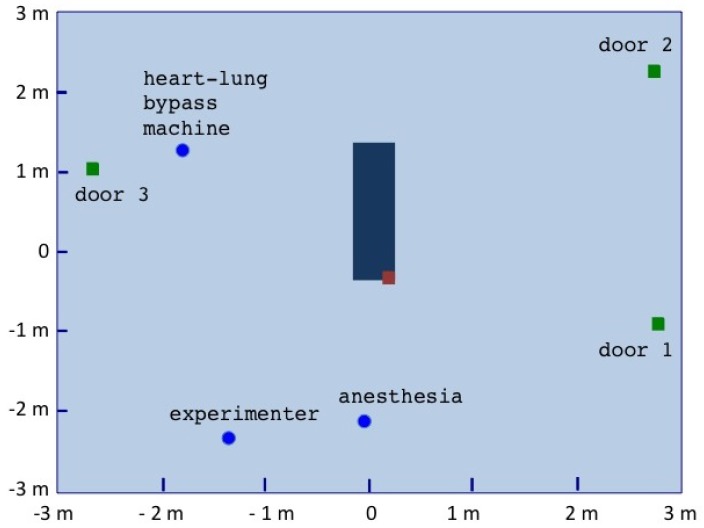
The performed mapping of the operating room seen in Figure 1. The brown square on the operating table locates the right shoulder of the patient.

A cartography of the room was initially performed, right after the calibration of the motion capture system, in order to 2D locate doors, the surgery table, and specific zones such as the experimenter desk or the anesthesia equipment using markers that were then removed (Figure 4).

During each intervention, identified by an ID number, the following information was recorded and saved on a hard disk:

- textual comments from the operator through a guided user interface (GUI)
- static 3D positioning of key points in the surgical operating room: table corners, doors, etc.
- dynamic 3D positions of the objects tracked by the VICON
- dynamic data from the three inertial units installed on each door *i*

An experimenter (operator) was sitting in the operating room during the intervention, as illustrated in Figure 2.

### 2.4. Data Processing

Data from the intervention was processed offline several days after the experiment. The post-processing software was developed in Python. Raw input data were processed and combined in order to extract for each sample the number of persons present in the room, their position, and the status of the doors.

#### 2.4.1. Door Event Extraction

The IMU raw data were processed in order to determine the door status: open, closed, at each time instant.

The FOX multi-points data system logged the raw values of magnetometer 3D magnetometer, 3D accelerometer, and 3D gyroscope each 200 ms.

We used the norm of the three-axis signals for each type of sensor. This allowed measuring the status of the doors independently of the orientation of the sensor positioned on the door.

In the absence of any magnetic disturbances, we used the magnetometer signals’ norm with a basic threshold detection to identify the two states of the corresponding door: open and closed throughout the intervention. Before starting the data collection, we proceeded to a calibration in order to identify the static norm values corresponding to open and closed door states. These values allowed us to define the corresponding thresholds.

For a few experiments, we observed the presence of magnetic disturbances, and we had to use a basic peak detection of the accelerometer signals (translating door) or accelerometers and gyroscopes signals (pivoting doors).

#### 2.4.2. Staff 3D Positions Pre-Filtering

Kinematic raw data recorded during the intervention consisted of the 3D positions ($x_{i},y_{i},z_{i}$) of the objects detected by the motion capture device throughout the intervention duration.

The first step in the process was to filter the data and remove outliers.

Depending on data quality, different filters were applied and combined to pre-filter raw data:

- filter by pieces: if the time interval between two detected positions of the object exceeded a predefined duration, the trajectory was segmented.
- minimum points per time window: if the number of detected positions of the object points in a predefined time interval was less than a predefined number, the point was considered isolated and removed.
- 1D outliers in the different axes (*x*, *y*, *z*): the distance to the median was computed for each 1D point in a sliding time window; if the distance exceeded a determined value, the point was removed.
- norm outliers: the distance to the median was computed for the norm of each 2D point (*x*, *y*) in a sliding time window; if the distance exceeded a determined value, the point was removed.

#### 2.4.3. Combination of Staff 3D Positions and Door Events

After data pre-filtering, the 3D trajectories of the individual caps were correlated to the door events. Each time an object was detected for the first time, its position was compared to the door location from the cartography information. If the object was spatially close to a door $Ddist_{min}$ and if an opening/closing event of the door was detected in a pre-defined time interval $Dtime_{min}$, then the event was associated with the object as the first point of its presence in the room. Similarly, if the object “disappeared”, the spatial distance to the closest door and the time distance to the last opening/closing event were analyzed to decide whether the object had left the room or if it was non-visible, but still present in the room. This allowed us to cut the trajectories of the objects in between entering/leaving the room events. Once segmented, each sub-trajectory was filtered with the same filters described in Section 2.4.2.

Within a given sub-trajectory, if an object position was missing, if the distance separating this position to the previous estimated position of the same object was inferior to $Odist_{min}$, and if the time delay between the two appearances of the object was less than $Otime_{min}$, an interpolation was applied to estimate the missing positions. If the distance or the time delay between the two appearances of the object was too important, the experimenter could also reconstruct data based on quantitative or qualitative information recorded during the intervention or on knowledge. For instance, if a position reached a zone in the limit of the MOCAP acquisition volume and appeared some meters further along the zone and if no door was located in the vicinity, the experimenter could manually force the interpolation between the last and next positions or impose a trajectory.

For each object, a file was generated with the 2D positions ($x_{i},y_{i}$) and the corresponding object status at each time instant: ID_targetname.csv. The status informs about the absence or presence of the object in the OR. In the situation when the algorithm did not converge to a decision on the status, the object position was considered as undetermined for this time instant. If the object was present, information was added to ensure traceability: position directly extracted from the raw data, position reconstructed (interpolated or forced), object OR entry/exit event.

### 2.5. Ethics and Risk Assessment

The protocol received the approval of the Institutional Review Board of Paris North Hospitals, Paris 7 University AP-HP (ID 11-113, 6 April 2012). The system was presented to surgical and anesthesiology teams in each participating center and OR [6]. An agreement was obtained from the head of surgery, the head of anesthesia, and the executive board of each participating center. Included patients were systematically informed by an information letter. Additionally, we collected the consent of each OR member participating in the study. A risk assessment was performed by: (1) testing during one month all the equipment by simulating an operating room in a classic room; (2) testing the device by simulating surgical procedures in a real operating room without patients; (3) testing the system in real conditions during two pilot surgical procedures with an experimented surgical team. Surgical team members were interviewed to identify possible issues. The system was considered non-disruptive of the normal flow of surgical procedures and did not present any risk for patient.

## 3. Results

We first illustrate the results on a representative intervention and then present the general results obtained on the 30 interventions.

### 3.1. Illustrative Intervention

Observations from the cardiac intervention ID22 are presented and detailed. Data were recorded during the five-hour period between the patient entry in and exit from the operating room. In total, 12 Tracker objects were detected, i.e., 12 different persons wearing caps. The room was equipped with three doors.

**Figure 5:**
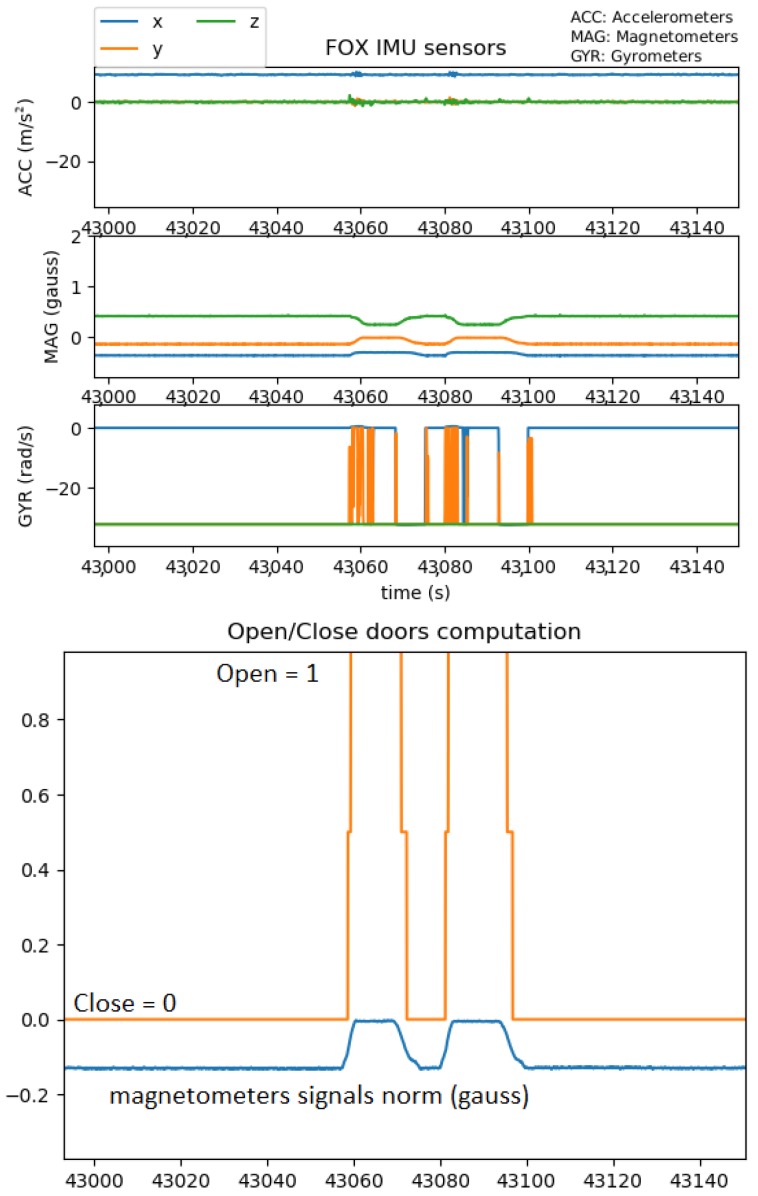
**Top**: Raw data of one IMU of Door 1; **Bottom**: Corresponding door opening/closing events.

For each of the three doors, raw IMU data (Figure 5 top) were processed in order to detect opening/closing events (Figure 5 bottom).

**Figure 6:**
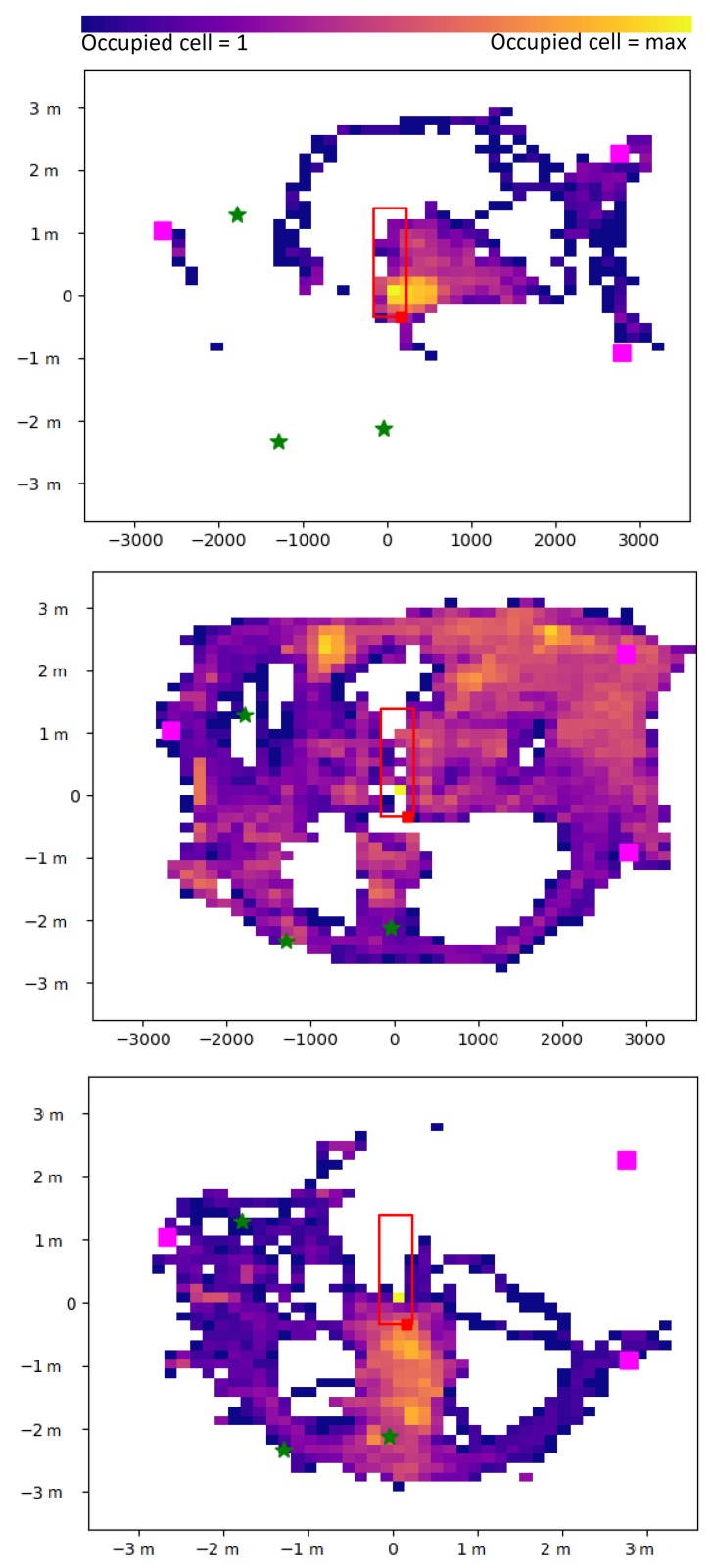
2D displacements of (**top**) the surgeon ($surgeon_{1}$), (**middle**) the operating room nurse ($nurse_{1}$), and (**bottom** ) the anesthetist nurse ($anesthetist_{1}$) over the whole cardiac surgery intervention.

**Figure 7:**
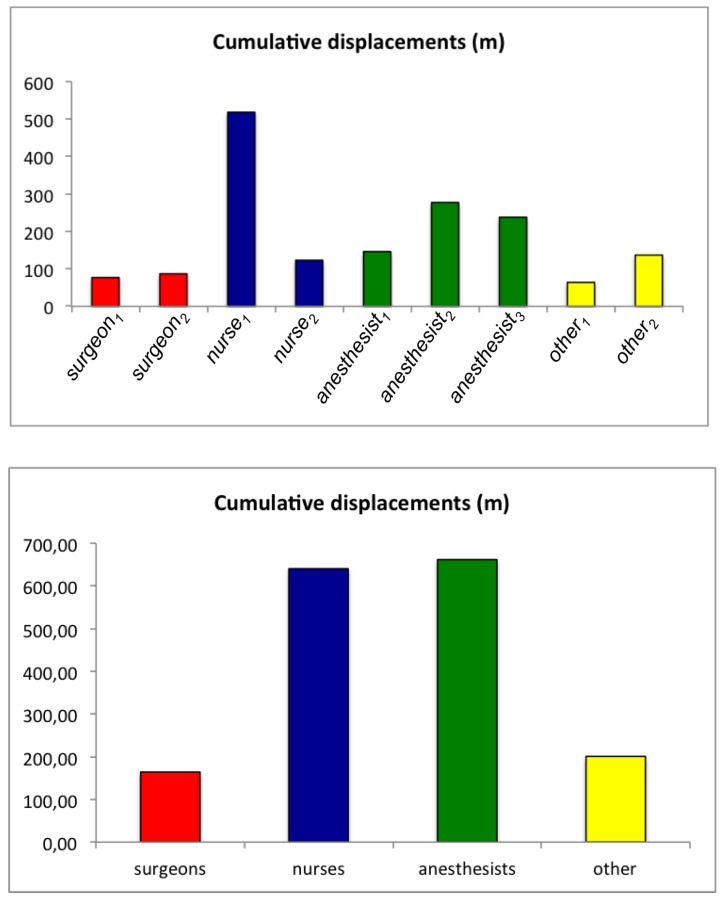
Cumulative distance traveled for the different categories of detected objects during the intervention (ID 22).

After MOCAP data processing, the center position of the object, corresponding to the cap displacements of one individual all along the whole surgery, could be assessed (Figure 6). In this example, we can clearly observe that the surgeon ($surgeon_{1}$) remained mainly in the vicinity of the operating table zone, whereas the nurse ($nurse_{1}$) was moving all over the room, and the anesthetist ($anesthetist_{1}$) was mainly staying in the zone behind the patient’s head. Quantitative assessment of the cumulative displacements over the entire intervention can also be analyzed. In Figure 7, for each detected object, the total distance traveled is plotted. This distance is clearly linked to the corresponding staff category to which the object belongs (surgeon, nurse, anesthetist, other).

**Figure 8:**
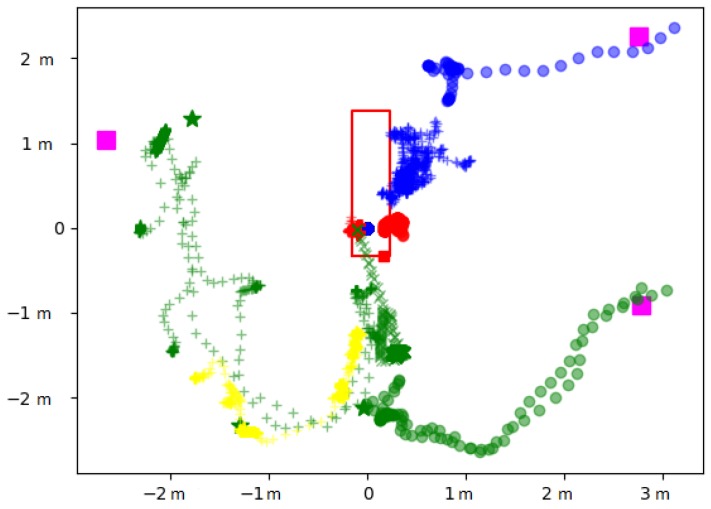
2D displacements of nine individuals present in the operating room from time 11 h 55 m 50 s to 11 h 58 m 20 s. $surgeon_{1}$ (o), $surgeon_{4}$ (+), $nurse_{1}$ (o), $nurse_{2}$ (+), $anesthetist_{1}$ (o), $anesthetist_{2}$ (+), $anesthetist_{4}$ (x), $other_{1}$ (+), $other_{1}6$ (o).

The displacements can be analyzed over specific periods to better understand the behaviors, as illustrated in Figure 8. In this focus, from time 11 h 55 m 50 s–11 h 58 m 20 s, during the microbiological air count procedure notified manually at 11 h 55 m 6 s by the operator, we could track all the displacements finely. For example, we can observe that $anesthetist_{2}$ moved to the heart-lung bypass machine (see Figure 4 for the localization) to perform the corresponding procedure.

**Figure 9:**
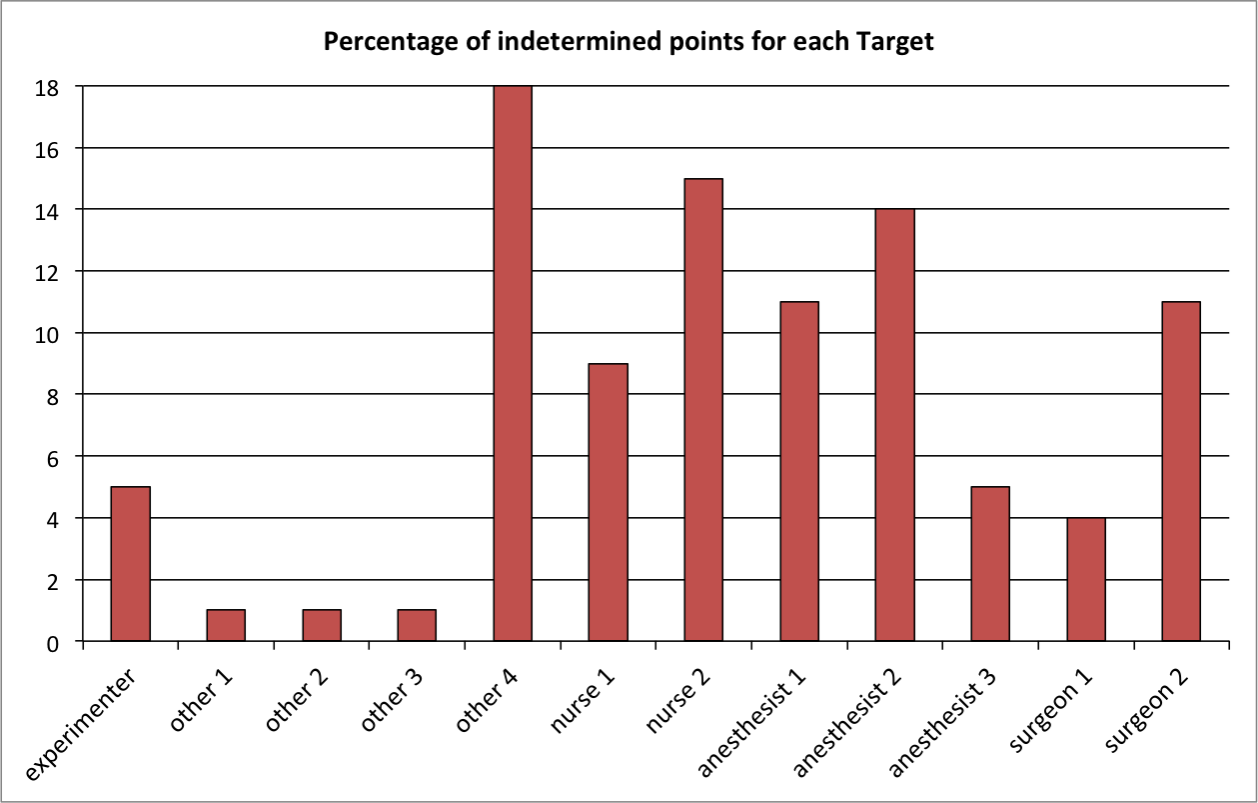
Percentage of undetermined points in the position reconstruction of each of the 12 detected objects during the intervention (ID 22).

The quality of the individual displacements reconstruction can be assessed through the percentage of undetermined points for each tracked object (Figure 9). For this specific intervention, which lasted five hours, the average of the undetermined samples over all 12 detected objects was 8%, and for each individual object, this score was lower than 20%.

### 3.2. General Analysis

We have analyzed data of 30 surgical interventions (14 in cardiac surgery and 16 in orthopedic surgery) at eight different clinic sites. Table 1 summarizes the reconstruction quality obtained for the different interventions.

**Figure 10:**
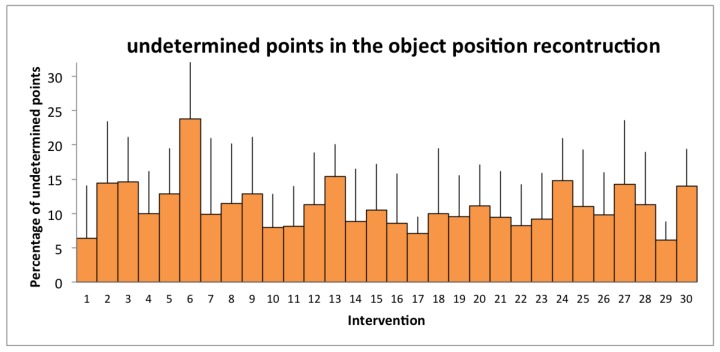
Percentage of undetermined points in the position reconstruction of the different objects of each intervention.

For a total of 119 h of data recorded, 334 object trajectories were analyzed. The quality of the individual displacement reconstruction can be assessed through the average percentage of undetermined points (Figure 10): 10.6% over the 30 interventions, which did not exceeded 24% for each individual intervention. These data were acquired, analyzed, and combined with other information to study the impact of staff behaviors on infectious risk in surgery [8].

## 4. Conclusions

We have designed a complete setup and methodology to assess continuously the number and category of persons present inside an operating room (OR), quantify their displacements inside the room, and identify door opening and closing events. We have developed and installed in eight different sites a setup composed of one motion capture device associated with an inertial measurement unit wireless network. Each individual entering the OR was asked to wear a cap equipped with four reflective markers. Offline signal processing of the acquired data allowed us to estimate the frequency of door openings during orthopedic and cardiac surgery interventions from incision to closure stages and the number of persons present in the room at each instant, and the accumulated distance traveled by the staff individuals could be estimated. Even though the operating room environment is not well adapted to motion capture technology, we have managed to estimate the displacements of 334 individuals during 30 different surgical procedures successfully. On average, the presence of a given individual in the OR was not possible to determine 10% of the time.

For each surgical procedure, microbiological air counts, continuous particle counts, and one bacteriological sample of the wound before skin closure were also performed. Statistics was then performed in order to assess the impact of staff behavior inside the OR on the infection risk [8]. The median frequency of door openings from incision to closure was found to be independently associated with an increased $log_{10}0.3$-µm particle and air microbial count. The number of persons and the cumulated movements by the surgical team were strongly associated with $log_{10}0.3$-µm particle counts. This study has demonstrated a previously missing association between intraoperative staff movements and surrogates of the exogenous risk of surgical site infection. Restriction of staff movements and door openings should be considered for the control of the intraoperative exogenous infectious risk.

## Figures and Tables

**Table 1:** From left to right: (1) intervention ID number, (2) OR location, (3) Surgery type (orthopedic or cardiac), (4) duration of the intervention, (5) number of detected objects, (6) number of doors, (7) number of objects discarded because of an insufficient detection rate (undetermined (u) >50%), (8) mean/std value of the percentage of undetermined status of the different objects.

ID	Site	Surgery	Duration h:mm	Nb Detected Objects	Nb Doors	Nb Discarded Objects	% Undet. Mean ± std
1	16	ort	2:33	9	1	1	6.4 ± 7.7
2	16	ort	2:33	7	1	0	14.4 ± 9
3	16	ort	2:32	11	1	2	14.6 ± 6.6
4	16	ort	3:17	8	1	3	10 ± 6.2
5	07	ort	4:02	11	2	3	12.9 ± 6.6
6	07	ort	3:19	13	2	7	23.8 ± 18.3
7	15	ort	2:30	10	2	0	9.9 ± 11.1
8	15	ort	2:50	10	2	1	11.5 ± 8.7
9	15	ort	2:55	10	2	1	12.9 ± 8.3
10	15	ort	2:25	13	2	0	8 ± 4.9
11	15	ort	2:12	14	2	0	8.1 ± 5.9
12	06	ort	1:58	9	1	0	11.3 ± 7.6
13	06	ort	1:56	12	1	0	15.4 ± 4.7
14	06	ort	2:06	11	1	1	8.8 ± 7.7
15	06	ort	2:31	11	1	0	10.5 ± 6.7
16	06	ort	2:35	10	1	0	8.6 ± 7.2
17	13	car	4:39	9	1	0	7.1 ± 2.4
18	13	car	6:22	10	1	0	10 ± 9.5
19	13	car	6:52	8	1	0	9.5 ± 6.1
20	17	car	5:06	14	3	1	11.1 ± 6
21	17	car	6:51	13	3	0	9.5 ± 6.7
22	17	car	4:42	12	3	1	8.2 ± 6.1
23	17	car	4:11	11	3	0	9.2 ± 6.7
24	03	car	4:50	12	1	1	14.8 ± 6.2
25	03	car	4:52	11	1	2	11 ± 8.3
26	03	car	6:51	13	1	1	9.8 ± 6.2
27	03	car	5:53	10	1	0	14.3 ± 9.3
28	03	car	5:30	15	1	1	11.3 ± 7.7
29	01	car	4:04	12	4	1	6.1 ± 2.7
30	01	car	6:14	15	4	1	14 ± 5.4

## References

[1] Lidwel O., Lowbury E., Whyte W., Blowers R., Stanley S.J., Lowe D. (1982). Effect of ultraclean air in operating rooms on deep sepsis in the joint after total hip or knee replacement: A randomised study. Br. Med. J..

[2] Tammelin A., Hambraeus A., Stahle E. (2001). Routes and sources of Staphylococcus aureus transmitted to the surgical wound during cardiothoracic surgery: Possibility of preventing wound contamination by use of special scrub suits. Infect. Control. Hosp. Epidemiol..

[3] Lynch R., Englesbe M., Sturm L., Bitar A., Budhiraj K., Kolla S., Polyachenko Y., Duck M.G., Campbell D.A. (2009). Measurement of foot traffic in the operating room: Implications for infection control. Am. J. Med. Qual..

[4] Young R., O’Regan D. (2010). Cardiac surgical theatre traffic: Time for traffic calming measures?. Interact. Cardiovasc. Thorac. Surg..

[5] Birgand G., ASaliou P., Lucet J.C. (2015). Influence of Staff Behavior on Infectious Risk in Operating Rooms: What is the Evidence?. Infect. Control Hosp. Epidemiol..

[6] Birgand G., Azevedo Coste C., Toupet G., Pissard-Gibollet R., GrandBastien B., Fleury E., Lucet J.C. (2014). Attitudes, risk of infection and behaviours in the operating room (the ARIBO Project): A prospective, cross-sectional study. BMJ Open.

[7] Jiménez A.R., Seco F., Peltola P., Espinilla M. Location of Persons Using Binary Sensors and BLE Beacons for Ambient Assitive Living. Proceedings of the 2018 International Conference on Indoor Positioning and Indoor Navigation (IPIN).

[8] Birgand G., Timsit J.F., Azevedo Coste C., Pissard-Gibollet R., Lucet J.C. (2019). Motions capture system to assess intraoperative staff movements and door openings: Impact on surrogates of the infectious risk in surgery. Infect. Control Hosp. Epidemiol..

[9] Andries M., Simonin O., Charpillet F. (2016). Localization of Humans, Objects, and Robots Interacting on Load-Sensing Floors. IEEE Sens. J..

[10] Alsinglawi B., Liu T., Nguyen Q.V., Gunawardana U., Maeder A., Simoff S. (2016). Passive RFID Localisation Framework in Smart Homes Healthcare Settings. Stud. Health Technol. Inform..

[11] Sevrin L., Noury N., Abouchi N., Jumel F., Massot B., Saraydaryan J. Characterization of a multi-user indoor positioning system based on low cost depth vision (Kinect) for monitoring human activity in a smart home. Proceedings of the 2015 37th Annual International Conference of the IEEE Engineering in Medicine and Biology Society (EMBC).

[12] Cappozzo A., Croce U.D., Leardini A., Chiari L. (2005). Human movement analysis using stereophotogrammetry: Part 1: Theoretical background. Gait Posture.

[13] Ancillao A., van der Krogt M.M., Buizer A.I., Witbreuk M.M., Cappa P., Harlaar J. (2017). Analysis of gait patterns pre- and post- Single Event Multilevel Surgery in children with Cerebral Palsy by means of Offset-Wise Movement Analysis Profile and Linear Fit Method. Hum. Mov. Sci..

[14] Ancillao A., Savastano B., Galli M., Albertini G. (2017). Three dimensional motion capture applied to violin playing: A study on feasibility and characterization of the motor strategy. Comput. Methods Programs Biomed..

[15] Tran D., Sakurai R., Yamazoe H., Lee J. (2017). Phase Segmentation Methods for an Automatic Surgical Workflow Analysis. Int. J. Biomed. Imaging.

[16] Watanabe E., Satoh M., Konno T., Hirai M., Yamaguchi T. (2016). The Trans-Visible Navigator: A See-Through Neuronavigation System Using Augmented Reality. World Neurosurg..

[17] Park J., Kim K., Kuh S., Chin D., Kim K., Cho Y. (2012). Spine surgeon’s kinematics during discectomy according to operating table height and the methods to visualize the surgical field. Eur. Spine J..

[18] Takayasu K., Yoshida K., Mishima T., Watanabe M., Matsuda T., Kinoshita H. (2018). Analysis of the posture pattern during robotic simulator tasks using an optical motion capture system. Surg. Endosc..

